# The influence of achievement motivation on college students’ employability: A chain mediation analysis of self-efficacy and academic performance

**DOI:** 10.3389/fpsyg.2022.972910

**Published:** 2022-10-06

**Authors:** Xiang Li, Ruihui Pu, Nutteera Phakdeephirot

**Affiliations:** ^1^Rattanakosin International College of Creative Entrepreneurship, Rajamangala University of Technology Rattanakosin, Nakhon Pathom, Thailand; ^2^School of Foreign Languages and Cultures, Panzhihua University, Panzhihua, Sichuan, China; ^3^Faculty of Economics, Srinakharinwirot University, Bangkok, Thailand

**Keywords:** achievement motivation, employability, self-efficacy, academic performance, college students, higher education

## Abstract

Employability of college students has been attached great importance by higher education institutions, employers, and governments because college graduates are the strategic human resource for the sustainable growth of universities, organizations, and countries across the world. It is also receiving growing attention from academic community. This study aimed to examine the psychological mechanism that impacts college students’ employability. It adopted an empirical approach by collecting data from 646 final-year students from 9 universities in the mainland of China. SPSS 25.0 was used for description, correlation, and regression analysis. AMOS 24.0 was utilized for path analysis. Model 6 Bootstrap method of PROCESS Version 3.5 was adopted for mediation analysis. The results showed that achievement motivation positively predicted self-efficacy, academic performance, and employability among undergraduates. Participants’ self-efficacy did not significantly impact their employability or play a mediating role in the relationship between achievement motivation and employability, while academic performance was a significant mediator of this association. Self-efficacy and academic performance served as chain mediators in the prediction of achievement motivation on college students’ employability. After controlling gender and family residence, achievement motivation still had significant and positive impact on employability of college students. This research made several noteworthy contributions to the existing studies on college students’ employability and provided insight for practitioners in strengthening their employability through these psychological constructs.

## Introduction

A growing number of students are graduating from higher education institutions across the world in recent years. On the one hand, converting these potential human resources into available talents to satisfy social needs is a significant way to promote economic development and social progress. These newly graduated talents can ensure the competitive advantage of organizations and innovation-driven development of nations. On the other hand, mounting pressure is laid on the shoulders of employers and governments to provide sufficient job positions for them. The rage of COVID-19 pandemic worsened the worrying situation due to economic slowdown (Leslie and Wilson, 2020) and financial uncertainties (Volenec et al., 2021). As a result, it became increasingly important for college students to enhance their employability to secure a job after graduation and contribute to sustainable economic growth.

Employability of college students has become a hot research field in academia in the past few years in various disciplines such as human resource management (Donald et al., 2019; Nimmi and Donald, 2022), educational management (Peng et al., 2021), psychology (Wang et al., 2021), sociology (Ingram and Allen, 2019), etc. Higher education institutions are striving to integrate employability developing programs into their educational system to ensure that the college students can be equipped with necessary knowledge, skills, attributes, and behaviors for a successful transition from campus to workplace (Yawson and Yamoah, 2020). Scholars have been making continuous academic efforts from the perspectives of definition (Harvey, 2001), influencing factors (Nimmi and Donald, 2022), models (Dacre Pool and Sewell, 2007), and other aspects of undergraduates’ employability to help tertiary institutions improve the performance of talent cultivation.

Among these existing studies, achievement motivation was tested to be positively associated with employability (Diao, 2015). Achievement motivation is an intrinsic desire that motivates people to develop their capabilities to successfully achieve the dreams they pursue in their daily lives and in a variety of activities (Suny and Suardiman, 2019). Motivation theories in contemporary education-related studies embody a variety of properties, such as goals, needs, desires, emotions, values, and interests (Wang et al., 2020). Motivation has a significant correlation with individuals’ behavior, to be specific, positive motivation is able to stimulate good behavior, while negative motivation holds back such behavior (Chiang et al., 2018). Achievement motivation has an impact on students’ success and excellence in the process of academic study (Nofrizal et al., 2020), which, in turn, influences their employment prospects upon graduation (Kamaliah et al., 2018). Prior studies have confirmed that achievement motivation significantly impacts the employability of college students, but only a few studies investigated the underlying mechanism of such influence. More specifically, no studies have been found to examine the path from achievement motivation to undergraduates’ employability through the chain mediating effect of self-efficacy and academic performance.

According to the social cognitive theory that interprets how the mechanisms of self-influence and self-regulation stimulate and govern human behavior (Owusu-Agyeman and Fourie-Malherbe, 2019), self-efficacy is identified as a dominant behavior determinant (Lee et al., 2018). It reflects an individual’ beliefs about his or her ability to take actions, accomplish tasks, and reach goals under different situations (Bandura, 1986). Previous research revealed that achievement motivation and self-efficacy were significantly correlated (Rui et al., 2011; Sabti et al., 2019), and self-efficacy was tested to have significant and positive influence on employability (Lian et al., 2021). However, the majority of these studies concentrated on exploring the impact of self-efficacy on employability through the mediation of achievement motivation (Wang et al., 2022). Less attention was paid to examine the influence of achievement motivation on employability through the mediation of self-efficacy among college students.

Additionally, achievement motivation from social cognitive perspective comprises of expectancy and value components in a broad sense, and previous studies based on such social cognitive model found that students’ motivational beliefs had significant and positive predicting effect on their academic performance (Muenks et al., 2018; Steinmayr et al., 2019). In the meanwhile, several studies showed a significant positive relationship between students’ self-efficacy and their academic performance (Shoval et al., 2021; Macakova and Wood, 2022). In terms of the research objects of the current study, students’ academic performance at the university was tested to have a significant positive contribution to their employment outcomes (Chhinzer and Russo, 2018; Tentama and Abdillah, 2019). As a result, it could be of great theoretical and practical importance to explore the functioning mechanism through which achievement motivation, self-efficacy, and academic performance influence undergraduates’ employability, which has not yet been empirically investigated.

By conducting a questionnaire survey among undergraduates in the mainland of China, this study aims to examine how achievement motivation impacts self-efficacy and subsequently influences academic performance and employability under the psychological mechanism of social cognitive theory. The findings could provide theoretical insight and empirical evidence for the research on the correlation between crucial psychological constructs and undergraduates’ employability. The results would also offer feasible positive interference for higher education institutions to enhance the employability of their students.

## Literature review and hypothesis development

### Employability

Employability is a combination of knowledge, skills, thought, and personal traits that improve an individual’s prospects of finding and maintaining jobs that are both satisfying and profitable to them (Dacre Pool and Sewell, 2007). It refers to the integration of traits such as capability, personality, desire, and social resources to ensure employment, including the knowledge and skills a person possesses in the course of career development, and a variety of extensive adjustments to the work environment (Yorke, 2006). It enables people to effectively accomplish work assignments by giving full play to their career-related abilities with proactive occupational behaviors (Schreuder and Coetzee, 2011).

Since the subjects of the current research are students in higher education institutions, the specific definition of college students’ employability can be viewed as a set of capabilities that assist students to find jobs upon graduation and attain success in the process of their career development, such as occupational knowledge, skills, self-regulation, communication skills, and interpersonal relationship (Harvey, 2001). It reflects the integrative abilities to accomplish satisfying work performance derived from learning ability and has turned into a critical factor for the career success of college graduates. Cultivating students’ career-related competencies and traits has become a core strategy for the transformation and development of higher education in many countries (White et al., 2021). Strong employability skills can empower college students with competitive advantages in the job market and ensure their success in the workplace of the future (Millican and Bourner, 2011).

### Achievement motivation

Achievement motivation refers to the intrinsic drive within individuals that motivates them to accomplish important and meaningful tasks and leads them to excel at what they do (Ye and Hagtvet, 1992). It is a complicated construct that includes several components such as self-perceived competency, task values, goals, and motives (Steinmayr et al., 2019), which refers to the kind of motivation that drives individuals for excellent performance, competitive advantage, perseverance, and growing efforts in fulfilling activities or tasks (Smith et al., 2019). As an internalized competency, it allows individuals to mobilize and manage their social and physical resources in a manner that improves performance and builds personal skills for success (Werdhiastutie et al., 2020). Student achievement motivation theory holds that students with this psychological trait are fully motivated to achieve goals successfully, develop their own abilities more confidently, and avoid failure in a variety of changing situations (Ishihara et al., 2018). Prior research found that achievement motivation had significant and positive correlation with the attainment of employability skills (Diao, 2015) because it can implant a stronger desire into individuals to strive for success and alleviate fear of failure (Tanjung and Musa, 2021). Individuals with strong achievement motivation have higher possibility to be accepted by job vacancies in the social contexts (Huang et al., 2022). Hence, the following hypothesis was put forward:

*H1*: Achievement motivation can positively predict employability of college students.

### Self-efficacy

Self-efficacy refers to individuals’ beliefs that they can perform a specific behavior and achieve anticipated outcomes (Bandura, 1977). It plays an important part in influencing people’s confidence in their ability to take actions and persevere under adversity (Yang et al., 2021). Self-efficacy leads individuals to believe that they have the capability to make productive use of motivational and cognitive resources to achieve the ultimate effect of the expected actions (Luthans, 2002). It is an important behavior-oriented factor that can affect students’ determination of learning objectives, choice of learning tasks, persistence of learning activities, and attribution of learning results.

#### Achievement motivation and self-efficacy

Individuals with strong intrinsic motivation to finish tasks and achieve goals tend to have self-confidence in taking specific actions and reaching desired objectives. For example, Wei et al. (2013) conducted a quantitative study on 336 Chinese students in higher education institutions and found that achievement motivation was positively correlated to self-efficacy in career decision. Damrongpanit (2019) suggested that achievement motivation was significantly and positively related with students’ self-efficacy in mathematics performance according to the research on 2,205 students and 117 teachers in Thailand. The study of 100 undergraduate students from two Iraqi public universities revealed that achievement motivation exhibited a significant positive correlation with self-efficacy in English writing (Sabti et al., 2019). Therefore, H2 was proposed as follows:

*H2:* Achievement motivation can positively predict self-efficacy among college undergraduates.

#### Self-efficacy and employability

Self-efficacy has been widely studied in a variety of research fields because it was positively related to learning strategies (Mornar et al., 2022), job performance (Downes et al., 2021), occupational success (Chughtai, 2018), self-esteem (Ouyang et al., 2020), and commitment (Yang et al., 2021). It is positively correlated with job search behavior and plays a crucial role in the employment of college graduates. Wang et al. (2022) found that self-efficacy was positively related to the levels of employability among college students in a quantitative study across six provinces in the Chinese mainland. A higher level of self-efficacy tends to make students more confident in seeking jobs when they graduate (Lian et al., 2021) and more employable in the job market (Chow et al., 2019). Thus, Hypothesis 3 was proposed:

*H3*: There is a positive prediction of self-efficacy on college students’ employability.

#### Achievement motivation, self-efficacy, and employability

The previous studies revealed that achievement motivation had a positive impact on students’ self-efficacy, and higher level of self-efficacy led to stronger sense of employability of students in colleges and universities. It implied that self-efficacy may have a mediating effect in the relationship between achievement motivation and employability. As a result, the following hypothesis was proposed:

*H4*: Self-efficacy played a mediating role in the correlation between achievement motivation and employability among college students.

### Academic performance

Currently, academic performance has no universally agreed-upon definition in the research field of higher education. Many researchers believe that college students’ academic achievements are the sum of their learning results, behaviors, and attitudes during the period of higher education, mainly including college students’ behavioral performance and objective achievements (Choi, 2005; Poropat, 2009; Stajkovic et al., 2018). Chamorro-Premuzic and Furnham (2003) measured academic performance by overall exam marks and final-year project performance. O’Connor and Paunonen (2007) argued that academic performance includes the grades of exams, essays, and courses as well as grade point average (GPA) and classroom performance. In general, the majority of the studies used GPA as a measure and indicator of students’ academic performance (Endalamaw Yigermal, 2017; Eakman et al., 2019; Lou, 2021). For example, Gatzka and Hell (2018) conducted a meta-analysis on the correlation between six facets of openness and postsecondary academic performance with students’ official GPA from higher education institutions as the criterion for their academic performance. Jan et al. (2020) made correlation analysis between emotional intelligence, library anxiety, and academic achievement among college undergraduates with GPA as the indicator for academic performance. Based on the previous research, the current study takes GPA as the measurement for academic performance.

#### Achievement motivation and academic performance

Many researchers have found that students’ achievement motivation is closely and positively correlated with academic performance (She et al., 2019; Gamazo and Martínez-Abad, 2020). Various constructs of motivation such as perceived control, values, and self-perception can be used as a supplement to intelligence tests to positively predict the academic performance (Daniels and Dueck, 2022). Achievement motivation of students influences their psychological and behavioral characteristics such as hope of success, coping with failure, persistence in adversity, and willingness to take more challenging courses (Yeager et al., 2019; Kapasi and Pei, 2022), which ultimately have an impact on their academic performance. A study on 4, 290 medical students from 10 Latin American countries indicated that their motivation was closely associated with achieving good academic performance during their college years (Torres-Roman et al., 2018). Hence, the following hypothesis was proposed:

*H5*: Achievement motivation plays a positive role in academic performance for college students.

#### Academic performance and employability

Several studies confirmed that academic performance is linked to college students’ employability (Dong et al., 2019; Tentama and Abdillah, 2019). There is a statistically significant relationship between academic achievement and employability and a positive correlation between employers’ perceptions of graduates’ employability and academic achievement (Chhinzer and Russo, 2018). Pinto and He (2017) found that higher academic performance led to greater job suitability and employability skills among business graduates from Peking University in China. A study on the graduate recruiters and employers who are looking for job applicants in the United Kingdom context revealed that academic achievements significantly affected the perceived employability of college graduates (Byrne, 2022). Hence, Hypothesis 6 was put forward as follows:

*H6:* Academic performance has a positive impact on college students’ employability.

#### Achievement motivation, academic performance, and employability

Based on the above arguments, achievement motivation can positively predict students’ academic performance, and better academic performance result in higher level of employability, which indicated that academic performance may mediate the effect of achievement motivation on employability. Therefore, this study proposed the following hypothesis:

*H7*: Academic performance played a mediating role in the correlation between achievement motivation and undergraduates’ employability.

### Chain mediating effect of self-efficacy and academic performance

It has been found that students’ self-efficacy is significantly correlated with their academic performance (Shoval et al., 2021; Macakova and Wood, 2022). A meta-analysis showed that self-efficacy was an influential factor to improve academic performance in online learning environment (Yokoyama, 2019). College students’ self-efficacy was found to have a positive effect on their academic performance in online English classes in China in the midst of the COVID-19 pandemic (Chang and Tsai, 2022). A systematic literature review on 27 articles revealed that self-efficacy and academic performance had significant and positive relationship among Latino students in America across all levels of education and with different measuring instruments (Manzano-Sanchez et al., 2018). Thus, the following hypothesis was proposed:

*H8*: College students’ self-efficacy has a positive impact on their academic performance.

In the light of the social cognitive theory, human functioning relies on the interaction between personal behavior (actions, choices, and verbal statements), internal personal factors (beliefs, expectations, attitudes, and knowledge), and environmental factors (resources, family, other people; Bandura, 1986, 1997). Students who are highly motivated to strive for excellence would have higher level of self-efficacy (Li and Li, 2018), generate better academic outcomes (Liu et al., 2019), and become more employable in the labor market (Dong et al., 2019). As a result, Hypothesis 9 was put forward:

*H9*: Self-efficacy and academic performance play chain mediating roles in the relation between college students’ achievement motivation and their employability.

### Control variables

Previous studies on employability have taken gender as a control variable (De Cuyper and De Witte, 2011; Misra and Mishra, 2016; González-Romá et al., 2018). Cifre et al. (2018) highlighted the necessity to delve into employability from the perspective of gender. At the same time, Mamaqi et al. (2011) included economic sectors as a control variable in their study on employability. In the current research, economic sectors refer to the family background of the participants. To be more specific, it takes the family residence in rural or urban areas of the sample into the regression model to test the effect as a control variable because students from urban families are usually in more favorable financial situations than their rural counterparts (Yang, 2010).

## Materials and methods

### Sample and procedure

The study was conducted at nine universities in the mainland of China. The convenience sampling method was employed to recruit participants (Assari, 2019). The objectives of the study and the policies of anonymity and confidentiality were presented before the participants filled the questionnaire. At last, 646 final-year college students participated in the survey with valid questionnaire. Among these respondents, 38.1% (*N* = 246) were male students, and 61.9% (*N* = 400) were female undergraduates. In terms of the family background, 46.1% (*N* = 348) of them were from rural households, and 53.9% (*N* = 298) were from urban families. As for the branches of academic disciplines that they studied in the universities, 45% (*N* = 291) were enrolled in Arts, 21.7% (*N* = 140) in Engineering, 30.2% (*N* = 195) in Management, and 3.1% (*N* = 20) in Medicine.

### Measures

Achievement motivation was measured with the Chinese version of The Achievement Motive Scale (Gjesme and Nygard, 1970). It originally had two dimensions: hope for success and fear of failure. Previous studies found that The Achievement Motive Scale with 30 items was unable to guarantee an acceptable fit to a two-dimension model (Lang and Fries, 2006). The current study ultimately kept the factor of “hope for success” with seven items after conducting exploratory factor analysis (EFA) and confirmatory factor analysis (CFA).

The present research adopted the Chinese version of The Morgan-Jinks Student Efficacy Scale (Jinks and Morgan, 1999) to measure the construct of self-efficacy of college students. Two dimensions were included in this study: effort and context. Various studies have proved the validity and reliability of the scale in measuring students’ self-efficacy (Magogwe and Oliver, 2007).

College Students’ Employability Scale (He, 2019) was utilized to test undergraduates’ employability in this study. Four dimensions with 15 items in total were used: (1) application of knowledge; (2) teamwork; (3) communication and coordination; (4) self-learning; and (5) self-management.

**Figure 1:**
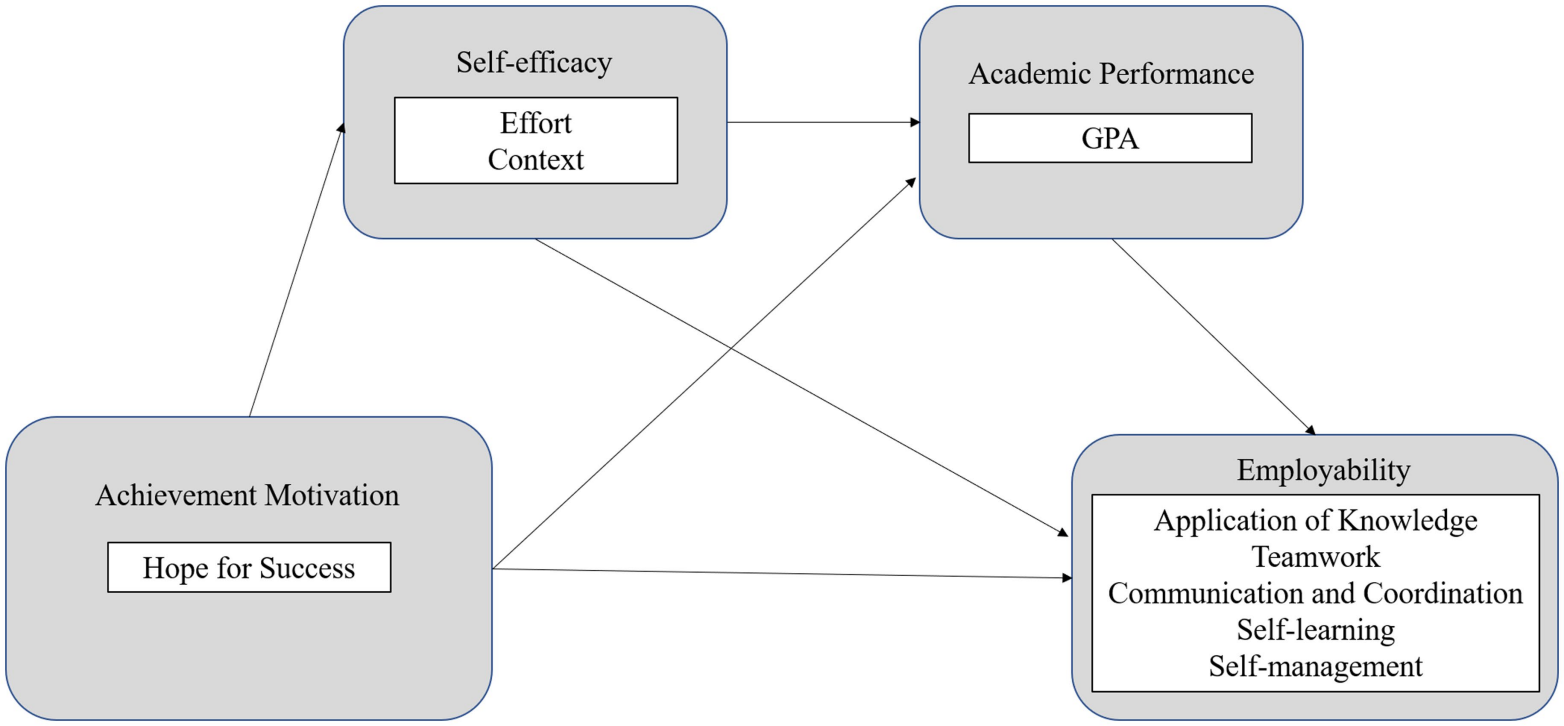
Hypothesized research model.

Figure 1 illustrates the hypothesized research model.

### Data analysis

This study conducted a three-step analysis of the data. First, SPSS 25.0 was utilized for explanatory factor analysis, descriptive analysis, correlation analysis between the studied variables, and regression analysis of the effect of the control variables. Second, AMOS 24.0 was used to conduct confirmatory factor analysis and path analysis to test the hypothesis. Third, PROCESS version 3.5 was adopted to test the mediating effect.

## Results

### Common method bias test

A common method deviation can occur when data is collected by self-report scales. Harman single-factor method was adopted in this study to examine the items of achievement motivation, self-efficacy, academic performance, and employability in an exploratory factor analysis. The results showed that there were six factors with initial eigenvalues greater than 1, which explained 65.049% of variance. Only 19.544% of the variance was explained by the principal factor, which is far less than the threshold of 40% (Liu et al., 2020). Therefore, Harman single-factor test indicated that common method bias was unlikely to be concerned in the present study.

### Validity and reliability of the scale

The overall scale and subscales used in the present study showed good reliability and validity. The values of Cronbach’s *α* and McDonald’s omega were used to evaluate the internal consistency of scales. The generally accepted threshold for the Cronbach’s *α* coefficient states that 0.9 ≤ *α* is deemed excellent, 0.7 ≤ *α* < 0.9 good, 0.6 ≤ *α* < 0.7 acceptable; 0.5 ≤ *α* < 0.6 poor; *α* < 0.5 unacceptable (Kulthanan et al., 2019). The Cronbach’s *α* value of the overall scale is 0.927. Table 1 shows that the values of each variable are greater than 0.8 and that of each subfactor greater than 0.7. The overall AM scale had a good reliability with Cronbach’s *α* coefficient of 0.862. The Cronbach’s *α* value of SE was 0.751, and that of its two subfactors are greater than 0.7. The entire EMP scale had an excellent reliability because the Cronbach’s *α* coefficient reached 0.950 and its five factors were all greater than 0.7. Values of McDonald’s Omega greater than 0.7 were deemed acceptable (Sharif Nia et al., 2018). As shown in Table 1, the coefficients of McDonald’s Omega were all greater than 0.7 except for the SMG because this dimension only had two items after CFA, while McDonald’s Omega estimate requires at least three items.

**Table 1:** Reliability and validity of the scale.

Scale	Dimension	Cronbach’s *α*	McDonald’s omega	CR	AVE
AM	AM	0.862	0.863	0.862	0.511
SE	EF	0.821	0.825		
	CON	0.738	0.714		
	SE-Total	0.790	0.742	0.827	0.549
EMP	AK	0.873	0.874		
	TM	0.883	0.885		
	CC	0.865	0.867		
	SL	0.790	0.812		
	SMG	0.788	*^a^		
	EMP-Total	0.950	0.949	0.929	0.545

AM, achievement motivation; SE, self-efficacy; EF, effort; CON, context; AP, academic performance; EMP, employability; AK, application of knowledge; TM, teamwork; CC, communication and coordination; SL, self-learning; SMG, self-management.

*^a^Omega cannot be estimated because the number of items for this dimension is 2.

In order to determine the construct validity, both convergent and discriminant validity were examined. A strong indication of convergent validity can be found when the average variance extracted (AVE) is at least 0.5 (Azizi and Khatony, 2019) as well as the composite reliability (CR) being greater than 0.7 (Kheirollahpour et al., 2020). Table 1 shows that values of AVE for each scale were greater than 0.5 and CR values are all above 0.8, which indicated a good convergent validity. A construct needs a higher AVE square root than the correlation coefficient between it and other constructs in order to have acceptable discriminate validity (Fornell and Larcker, 1981). As shown in Table 2, the square roots of achievement motivation, self-efficacy and employability are 0.715, 0.741 and 0.738, respectively. All constructs in this study had an AVE square root greater than their correlation with other constructs.

**Table 2:** Discriminate validity of the scale.

Items	AM	SE	EMP
AM	**0.715**		
SE	0.514^***^	**0.741**	
EMP	0.609^***^	0.653^***^	**0.738**

*** *p* < 0.001.AM, achievement motivation; SE, self-efficacy; EMP, employability.

### Differences in demographic variables

The demographic variables in this study included gender, family residence, and discipline. Independent-samples T-tests and One-way ANOVA were performed concerning achievement motivation, self-efficacy, academic performance, and employability among the participated final-year undergraduates.

Table 3 shows that self-efficacy scores of male students were significantly higher (*M* = 3.29, *SD* = 0.51) than their female counterparts (*M* = 3.15, *SD* = 0.44), *t* = 3.728, *p* < 0.001. Academic performance scores of the participants from urban families (*M* = 3.54, *SD* = 0.67) are significantly higher than those from rural ones (*M* = 3.37, *SD* = 0.67), *t* = 3.198, *p* < 0.01. The scores of employability were higher for those from urban families (*M* = 3.85, *SD* = 0.58) than those from rural households (*M* = 3.68, *SD* = 0.70), *t* = 3.282, *p* < 0.01. Employability scores of students majoring in engineering were higher (*M* = 3.87, *SD* = 0.66) than those in arts (*M* = 3.71, *SD* = 0.61), management (*M* = 3.83, *SD* = 0.66), and medicine (*M* = 3.51, *SD* = 0.60), *F* = 3.857, *p* < 0.01. Other than that, there were no significant differences in other variables between genders, family residences, and among different branches of academic discipline (*p* > 0.05).

**Table 3:** Basic description of the variables (*N* = 646).

Variable	Category	Achievement motivation	Self-efficacy	Academic performance	Employability
		*M* ± SD	*M* ± SD	*M* ± SD	*M* ± SD
Gender	Male	3.89 ± 0.65	3.29 ± 0.51	3.41 ± 0.70	3.80 ± 0.68
	Female	3.90 ± 0.49	3.15 ± 0.44	3.49 ± 0.67	3.75 ± 0.61
	*t*	−0.61	3.728^***^	−1.507	1.006
Family residence	Rural	3.88 ± 0.60	3.20 ± 0.49	3.37 ± 0.69	3.68 ± 0.70
	Urban	3.92 ± 0.52	3.20 ± 0.46	3.54 ± 0.67	3.85 ± 0.58
	*t*	0.902	0.219	3.198^**^	3.282^**^
Branches of academic discipline	Arts	3.87 ± 0.50	3.16 ± 0.45	3.47 ± 0.66	3.71 ± 0.61
	Engineering	3.98 ± 0.61	3.25 ± 0.53	3.48 ± 0.71	3.87 ± 0.66
	Management	3.89 ± 0.61	3.23 ± 0.48	3.43 ± 0.71	3.83 ± 0.66
	Medicine	3.79 ± 0.35	3.20 ± 0.34	3.40 ± 0.68	3.51 ± 0.60
	*F*	1.419	1.655	0.226	3.857^**^

** *p* < 0.01;

*** *p* < 0.001.

### Correlations between the variables

As shown in Table 4, the mean values of achievement motivation, self-efficacy, and employability and their subfactors of college students were all above 3. It suggested that the college students participated in this study basically had adequate levels of achievement motivation, self-efficacy, and employability. According to Table 4, achievement motivation had a significant correlation with undergraduates’ self-efficacy and employability (including their respective subdimensions). A noteworthy result is that although the overall self-efficacy was significantly related to employability in general, the context factor of self-efficacy only had a significant association with the application of knowledge dimension of employability. No significant correlation existed between the context factor and the other four dimensions of college students’ employability, while the effort factor of self-efficacy had significant and positive correlation with overall employability and its subfactors. Academic performance had significant and positive relation with achievement motivation, self-efficacy, employability, and their subdimensions except the context factor of self-efficacy.

**Table 4:** Means, standard deviations and correlations between variables.

Variable	*M*	SD	1	2	3	4	5	6	7	8	9	10	11
1. AM	3.90	0.56	1										
2. SE	3.20	0.47	0.308^**^	1									
3. EF	3.45	0.62	0.413^**^	0.718^**^	1								
4. CON	3.06	0.55	0.151^**^	0.892^**^	0.327^**^	1							
5. AP	3.46	0.69	0.487^**^	0.235^**^	0.468^**^	0.016	1						
6. EMP	3.77	0.64	0.556^**^	0.264^**^	0.512^**^	0.027	0.713^**^	1					
7. AK	3.56	0.79	0.483^**^	0.303^**^	0.474^**^	0.104^**^	0.565^**^	0.850^**^	1				
8. TM	3.95	0.68	0.505^**^	0.188^**^	0.416^**^	−0.015	0.678^**^	0.891^**^	0.663^**^	1			
9. CC	3.88	0.67	0.512^**^	0.218^**^	0.438^**^	0.012	0.666^**^	0.927^**^	0.705^**^	0.895^**^	1		
10. SL	3.74	0.74	0.506^**^	0.244^**^	0.495^**^	0.010	0.662^**^	0.916^**^	0.728^**^	0.741^**^	0.782^**^	1	
11. SMG	3.66	0.81	0.422^**^	0.198^**^	0.425^**^	−0.007	0.553^**^	0.791^**^	0.594^**^	0.572^**^	0.628^**^	0.783^**^	1

AM, achievement motivation; SE, self-efficacy; EF, effort; CON, context; AP, academic performance; EMP, employability; AK, application of knowledge; TM, teamwork; CC, communication and coordination; SL, self-learning; SMG, self-management.

** *p* < 0.01.

### Direct effect on employability

Before testing the hypotheses, this study conducted confirmatory factor analysis *via* AMOS 24.0 to examine the fitness of the proposed model. The results showed that the research model had a good fit (*χ*^2^/df = 3.599, GFI = 0.891, CFI = 0.926, NFI = 0.901, TLI = 0.915, SRMR = 0.0574, RMSEA = 0.063). AMOS 24.0 was also utilized to conduct path analysis to test the proposed hypothesis. As shown in Table 5, achievement motivation can positively predict employability at 0.001 level. Thus, H1 was supported. Achievement motivation can also positively predict self-efficacy at 0.001 level. Hence, H2 was supported. The path coefficient between self-efficacy and employability was not significant (*p* = 0.074 > 0.05). Therefore, H3 was rejected. Achievement motivation was tested to have positive prediction on academic performance at 0.001 level. Hence, H5 was supported. Academic performance was identified to have a positive impact on employability at 0.001 level. Therefore, H6 was supported. Self-efficacy was found to have a significant prediction on academic performance at 0.01 level (*p* = 0.009). Thus, H8 was supported.

**Table 5:** Test results of the proposed hypothesis.

Hypothesis	Path	Coefficient	SE	CR	Value of *p*	Test results
H1	Emp ← AM	0.260	0.035	8.552	***	Supported
H2	SE ← AM	0.308	0.032	8.220	***	Supported
H3	EMP ← SE	0.049	0.037	1.784	0.074	Rejected
H5	AP ← AM	0.458	0.044	12.751	***	Supported
H6	EMP ← AP	0.575	0.028	19.293	***	Supported
H8	AP ← SE	0.094	0.052	2.623	0.009	Supported

AM, achievement motivation; SE, self-efficacy; AP, academic performance; EMP, employability.

*** *p* < 0.001.

The regression analysis was conducted by SPSS Version 25.0. Gender and family residence were included as control variables in the regression equation. The result showed that achievement motivation of college students generated a significant and positive impact upon their employability after controlling the influence of the participants’ gender and family background (*β* = 0.551, *p* < 0.001).

### Chain mediating effect of self-efficacy and academic performance

This research adopted PROCESS Version 3.5 to examine the mediating effect of self-efficacy and academic performance on the association between achievement motivation and employability of college students. Model 6 was selected in PROCESS with Bootstrap samples of 5,000. Bias Corrected for Bootstrap CI method was chosen and the level of confidence for all confidence intervals was set at 95%. A statistically significant mediating effect occurs if the interval between BootLLCI and BootULCI does not include 0 (Preacher and Hayes, 2008).

After running the macro in SPSS 25.0, the following results were generated as shown in Table 6. It demonstrated that the total indirect effect of the prediction of achievement motivation on undergraduates’ employability was 0.34. The indirect effect of self-efficacy on the relationship between achievement motivation and college students’ employability was 0.017. The 95% confidence interval included 0 (BootLLCI = −0.005, BootULCI = 0.046), which indicated that the mediating effect is not statistically significant. Hence, H4 was rejected. Self-efficacy did not play a mediating role in the relationship between achievement motivation and undergraduates’ employability. The indirect effect of self-efficacy and academic performance on the correlation between achievement motivation and employability was 0.019. The 95% confidence interval did not include 0 (BootLLCI = 0.005, BootULCI = 0.038), which confirmed a significant mediating effect. Thus, H9 was supported in this research. Self-efficacy and academic performance had a chain mediating effect on the correlation between achievement motivation and undergraduates’ employability. The indirect effect of academic performance on the correlation between achievement motivation and employability was 0.303.0 and was not included in the 95% confidence interval (BootLLCI = 0.247, BootULCI = 0.367), which identified a significant mediating effect. Hence, H7 was supported. Academic performance functioned as a mediator in the correlation between achievement motivation and undergraduates’ employability.

**Table 6:** Mediating effect of self-efficacy and academic performance on the correlation between achievement motivation and employability.

Effect	Hypothesis	Model pathways	Indirect effect value	Boot SE	BootLLCI	BootULCI	Effect of the amount	Test results
Total indirect effect			0.340	0.030	0.284	0.403	53.13%	
Indirect effect path 1	H4	AM-SE-EMP	0.017	0.012	−0.005	0.046	2.70%	Rejected
Indirect effect path 2	H9	AM-SE-AP-EMP	0.019	0.008	0.005	0.038	3.00%	Supported
Indirect effect path 3	H7	AM-AP-EMP	0.303	0.031	0.247	0.367	47.43%	Supported

AM, achievement motivation; SE, self-efficacy; AP, academic performance; EMP, employability.

## Discussion

### Direct relationships

The current study confirmed that achievement motivation has a direct impact on employability of college students. The finding could provide theoretical and practical implications for both higher education providers and receivers. As for higher education administrators and teachers, they are propelled to pay high attention to students’ psychological factors to improve the overall quality of their graduates. Systematic integration of achievement motivation cultivation into educational plans and teaching curriculum to enhance students’ employability skills (Laguna-Sánchez et al., 2020) emerges as an urgent mission for the sustainability of higher education. In terms of college students, they need to be aware of the significant and positive influence of psychological attributes on their employment prospects and personal growth. Even if they encountered setbacks in academic activities or daily life on campus, they should stay motivated to achieve planned goals and acquire necessary knowledge and skills instead of “lying flat” (a recently widespread phenomenon among Chinese young people that rejects hard working and constant competition; Gao et al., 2022).

Many previous studies revealed that self-efficacy and achievement motivation were significantly correlated, but most of the studies examined the prediction of self-efficacy on achievement motivation (Liu et al., 2021; Huang et al., 2022). The present study added new evidence to the argument that achievement motivation had positive impact on self-efficacy at 0.001 level in the context of higher education. Students with intrinsic desire to attain achievements turned to be confident in their abilities in interacting with environmental factors and performing positive behaviors, which showed the valid application of social cognitive theory in China’s higher education sector.

Although a variety of prior studies found that self-efficacy had a significant and positive impact on employability of college students (Chow et al., 2019; Wang et al., 2022), the current research showed that the prediction of self-efficacy on employability was not statistically significant, which echoed with the research result by Coetzee and Oosthuizen (2012) who found self-efficacy was not significantly related to employability among adult learners enrolled in the open distance learning programs. Self-efficacy scores of the samples were relatively low (*M* = 3.20, SD = 0.47) in the current study, which indicated that the participants did not have much confidence in their devotion to studies and their capabilities to accomplish academic tasks. Two dimensions of self-efficacy were examined in the questionnaire survey. The mean score of “effort” dimension was 3.45, indicating that students generally believed that they were able to obtain satisfying grades when they worked hard. The “context” dimension had the lowest score (*M* = 3. 06, SD = 0.55) among all the 11 variables. It revealed that the participants had doubts about the purpose of receiving higher education and felt unsatisfied with the amount of attention received from their teachers. This might be related to the growing employment pressure on college students since they had a lot of difficulties in securing a job or even became unemployed after graduation (Wu et al., 2022). Furthermore, as a result of the expansion of enrollment of higher education in China (He et al., 2020), teachers have to face a large number of students in one class and teach several classes in one semester. For example, the minimum class size in Panzhihua University in Sichuan Province is 60 for compulsory courses and 120 for optional courses. The teachers there are normally assigned four different classes in each semester. As a result, teachers were unable to give sufficient attention to each student. At the same time, this research found that the context factor of self-efficacy was only positively correlated with the “application of knowledge” dimension of employability. It was not significantly related to any other factor of undergraduates’ employability. The “context” mainly refers to the external factors that influence a person’s belief in their own ability (Beck and Quinn, 2011). The findings of the current research revealed that students attached more importance to their “effort” in developing employability attributes. This could be partly explained by the advancement of information technology that enables students to have access to numerous educational resources (Li et al., 2021) and borderless social networking (Smirnov, 2019) across the world. As long as they are determined to develop skills, they would not have much impediment in reaching external educational resources.

Academic achievements that students obtained in college had a significant impact on their employability. On one hand, students with higher academic performance are often characterized by goal-orientation, perseverance in adversity, courage in taking daunting tasks, etc. (Lerang et al., 2019; Lam and Zhou, 2022), which are also highly-valued qualities in workplace and critical factors to ensure sustainable career success. On the other hand, higher academic performance indicates sound mastery of specific knowledge and professional skills in the given field, which are basic requirements in job responsibilities. However, higher education institutions in China are undergoing dramatic transformation. Many of them are becoming application-oriented and giving strategic priority to work-integrated learning, integration of production and education, integration of occupational qualifications, etc. (Zhang and Chen, 2022). As a result, they are running risk of attaching more importance to the needs from the industries than the actual academic performance of the students. The current study highlighted the significant and positive prediction of academic performance on undergraduates’ employability. It reminds the policy-makers and administrators to put the cultivation of students’ academic achievements at the first place to ensure the sustainable development of higher education.

### Mediated relations

The test results showed self-efficacy did not serve as a mediator in the relationship between achievement motivation and employability among the participants in this study. Achievement motivation was tested to have a significant prediction on self-efficacy and employability, but self-efficacy did not significantly enhance the impact of achievement motivation on undergraduates’ employability. This could be the result of the above-mentioned generally low self-efficacy scores of the participants and their uncertainties about the future after graduation. In the meanwhile, students with high level of achievement motivation would naturally mobilize cognitive, social, intellectual, and emotional resources (Zamroni et al., 2022) to attain set goals and become more employable in the labor market.

Academic performance was tested to mediate the relationship between achievement motivation and employability. College students are confronted with many challenges in their academic studies, campus life, and peer competition. Achievement motivation drives students to move forward in their learning activities, daily life, and acquisition of skills, which was confirmed to have a positive prediction on undergraduates’ employability in this study and previous research (Kamaliah et al., 2018). Stronger achievement motivation could keep them in a healthy mental state and equip them with positive attitudes, which, in turn, strengthens their study effects and improves their academic performance. Academic achievements are closely related to knowledge and skills that students acquired in higher education institutions, which lays a solid foundation for their professional competencies and career success (Thiele et al., 2018). As a result, academic performance enhanced the effect of achievement motivation on employability in the context of tertiary education.

### Chain mediating relation

The present study showed that self-efficacy and academic performance jointly mediated the impact of achievement motivation and employability of college students from the mainland of China. Based on the social cognitive theory, behavior, cognition, and environment are interconnected and mutually determined (Almuqrin and Mutambik, 2021). Students’ cognitive factors have a significant influence on their behavior. Their interactions with classmates, teachers, administrators, and other environmental factors reinforced their achievement motivation, self-efficacy, academic performance, and finally, the acquisition of necessary employability skills. Strong intrinsic motive to attain achievements stimulate positive self-efficacy and generate better academic outcomes, which effectively enhances their employability. The test results underlined the significance of developing students’ self-efficacy in the pedagogical endeavors in addition to enhancing their motive to succeed because self-efficacy determines individuals’ belief in performing behaviors and, in turn, affects their efforts and prospects in accomplishing specific tasks. When students are highly motivated and also believe in their ability to achieve the goals (Yang et al., 2021), they would be equipped with strong employability skills that are most sought after by employers.

## Conclusion

This study empirically examined the relationship between achievement motivation, self-efficacy, academic performance, and employability among undergraduates. Based on the previous findings in academic literature about employability, the present research proposed and tested the hypothesis by collecting data from different universities in China. The results showed that achievement motivation positively predicted self-efficacy, academic performance, and employability of college students. Self-efficacy was tested to have no significant impact on employability of the participants and play no significant mediating role in the correlation between achievement motivation and undergraduates’ employability, while academic performance significantly mediated such relationship. The findings showed that self-efficacy and academic performance played chain mediating roles in the prediction of achievement motivation on employability of college students. After controlling gender and family residence, achievement motivation still significantly and positively affects employability of college students.

The findings of this research add new understanding to the existing literature on college students’ employability. The study identified the chain mediating effect of self-efficacy and academic performance in the relationship between undergraduates’ achievement motivation and employability and found that self-efficacy had no significant direct and indirect effect on employability in this sample. It provides timely evidence that higher education institutions should give students’ academic performance top priority against the background of current application-orientation transformation. They also need to take immediate actions to make students realize the value and meaning of attending colleges to ensure the sustainability of higher education because students have insufficient confidence in finding employment after graduation. This study has several valuable implications for future practice of improving students’ employability for higher education providers since these graduates will be the most valuable assets of organizations, highly skilled knowledge workers in the labor market, and the main force in sustainable development.

## Research limitations and future research directions

Although the study contributed to the existing literature on employability of college students in a number of theoretical and practical ways, it does have several limitations that need to be considered in future studies. Firstly, the latest official data from the Ministry of Education of China released on 30 September 2021 showed that there are 1, 270 higher education institutions in Chinese mainland offering bachelor’s degrees with 12 different academic disciplines. Only 9 public schools and 4 academic disciplines were surveyed in the current research due to the limited length of time and available contacts. When respondents were recruited from different types of universities and different branches of academic disciplines, the results might be different from those in this study. As a result, it would be necessary to test the results with a wider sample of participants in order to come to more general conclusions about the relationship between achievement motivation, self-efficacy, academic performance, and employability among university students. Secondly, the study examined the employability of college students in terms of subdimensions such as application of knowledge, teamwork, communication, coordination, self-learning, and self-management. Other potential dimensions of undergraduates’ employability were not yet paid attention to in the current analysis. Future studies are encouraged to examine other aspects of college students’ employability to gain a more comprehensive assessment. Thirdly, this research relies largely on questionnaire as its main research approach. A qualitative method might be helpful in revealing the profound correlation between the studied variables and provide valuable interpretation of the research findings from different perspectives.

## Data availability statement

The raw data supporting the conclusions of this article will be made available by the authors, without undue reservation.

## Ethics statement

The studies involving human participants were reviewed and approved by the Institutional Ethics Committee of the School of Foreign Languages and Cultures at Panzhihua University (approval code: no. HRECA21-006 and date of approval: 8 November 2021). The patients/participants provided their informed consent to participate in this study.

## Author contributions

RP: conceptualization. XL and RP: data curation, investigation, and methodology. RP and NP: supervision and writing–review and editing. XL: writing–original draft. All authors contributed to the article and approved the submitted version.

## Funding

This research was funded by the Ph.D. Research Start-up Fund of Panzhihua University (no. 035200187).

## Conflict of interest

The authors declare that the research was conducted in the absence of any commercial or financial relationships that could be construed as a potential conflict of interest.

## Publisher’s note

All claims expressed in this article are solely those of the authors and do not necessarily represent those of their affiliated organizations, or those of the publisher, the editors and the reviewers. Any product that may be evaluated in this article, or claim that may be made by its manufacturer, is not guaranteed or endorsed by the publisher.
